# Discordance in decision-making between patients with advanced cancer and caregivers from the perspective of healthcare professionals in specialist palliative care: A focus group study

**DOI:** 10.1017/S147895152510120X

**Published:** 2025-12-22

**Authors:** Joshua Hernon, John Lombard, Suzanne Guerin, Hannah Featherstone, Norma O'Leary, Geraldine Foley

**Affiliations:** 1 Discipline of Occupational Therapy, School of Medicine, Trinity College Dublin, Dublin 2, Ireland; 2 School of Law, University of Limerick, Limerick, Ireland; 3 School of Psychology, University College Dublin, Dublin 4, Ireland; 4 Department of Palliative Medicine, Our Lady of Lourdes Hospital, Drogheda, Ireland; 5 Department of Palliative Medicine, Our Lady’s Hospice and Care Services, Dublin 6, Ireland; 6 Department of Palliative Care, St. James’s Hospital, Dublin 8, Ireland; 7 School of Medicine, Trinity College Dublin, Dublin 2, Ireland

**Keywords:** Specialist palliative care, advanced cancer, patient, caregiver, discordance

## Abstract

**Objectives:**

Patients with advanced cancer, their caregivers, and healthcare professionals can differ in their preferences for patient treatment and care. The objectives of this study were to (1) identify what healthcare professionals in specialist palliative care feel aids or challenges patients with advanced cancer and their caregivers to manage their discordance, and (2) decipher what is helpful or challenging for healthcare professionals themselves to manage discordance between patients with advanced cancer and their caregivers.

**Methods:**

A qualitative study was conducted comprising online focus groups with 19 healthcare professionals from different professions in specialist palliative care. Participants were purposively and snowball sampled, and recruited from specialist palliative care settings, including hospital, hospice, and community-based care. The data were member checked and analyzed using thematic analysis.

**Results:**

Trust and consistent communication between the patient, caregiver, and healthcare professional, were considered by participants as helpful for patients and caregivers to manage discordance. Emotional and psychological burden for both the patient and caregiver together with preexisting conflict between the patient and caregiver, were perceived as barriers for patients and caregivers to manage their discordance. Knowledge and expertise gained from practice combined with professional resilience and peer support enabled participants to help patients and caregivers navigate discordance. Relational conflict between the patient and caregiver combined with participants’ own uncertainty about ethical and legal connotations of helping the patient and caregiver resolve their differences, were barriers to helping the patient and caregiver manage their discordance.

**Significance of results:**

Interventions focused on assisting patients with advanced cancer in palliative care and their caregivers manage their differences in decision-making could serve to alleviate emotional burden for both the patient and caregiver. Healthcare professionals in specialist palliative care value the perspective of both patients with advanced cancer and their caregivers when helping them manage their discordance in decision-making.

## Introduction

Specialist palliative care for people with advanced cancer involves healthcare professionals working across inpatient, outpatient, and community-based settings, focused on treating complex symptoms to optimize quality of life for patients and caregivers (Gouldthorpe et al. 2023). Treatment decision-making for cancer patients and their caregivers in palliative care is challenging, particularly in cases of advanced cancer (Back 2020). Whilst some patients with advanced cancer make decisions about care with healthcare professionals without their caregivers, many patients also make decisions with their caregivers (Dionne-Odom et al. 2019; Laryionava and Winkler 2021). Patients with advanced cancer, their caregivers, and healthcare professionals often differ in their preferences for patient symptom management and end-of-life care (Mulcahy Symmons et al. 2023; Levine et al. 2024). Discordance between the patient with advanced cancer and their caregiver in decision-making about patient treatment and care can be influenced by preexisting conflict between the patient and caregiver or result in new conflict between them (Fagan et al. 2024; Foley et al. 2025).

Healthcare professionals in palliative care operate within ethical frameworks that directly impact how they facilitate patient decision-making (Lombard 2018). Differences between patients’ and caregivers’ preferences for palliative care mean that healthcare professionals need to be sensitive to the viewpoint of both the patient and caregiver and, in tandem, facilitate patient autonomy in decision-making (Gomez-Virseda et al. 2019; Houska and Loučka 2019). However, how healthcare professionals in specialist palliative care facilitate both the patient with advanced cancer and the caregiver when the patient and caregiver differ in their preferences for and/or decisions they make about care, is not well understood (Mulcahy Symmons et al. 2023). The primary objective of this study was to explore specialist palliative care healthcare professionals’ perspectives on discordance between patients with advanced cancer and their caregivers in the decision-making process for patient treatment and care. We sought to identify what healthcare professionals in specialist palliative care feel aids or challenges patients with advanced cancer and their caregivers manage their discordance, and what is helpful or challenging for health professionals themselves to manage discordance between patients with advanced cancer and their caregivers.

## Methods

### Study design

We conducted a qualitative study comprising focus groups with healthcare professionals in specialist palliative care to achieve our aims. We used a qualitative approach because we sought to be primarily inductive in our approach to better understand the phenomenon under study and to elicit responses that we could sufficiently probe into to capture the meanings and understandings that participants attached to their experiences (Green and Thorogood 2018). We worked from a framework in qualitative research that emphasizes the importance of relationality to ensure credibility and relevance (Timonen et al. 2024). We referred to the Standards for Reporting Qualitative Research (O’Brien et al. 2014) in conducting the research. Ethical approval to conduct the study was received from the School of Medicine Research Ethics Committee at Trinity College Dublin in October 2024 (ref 3581).

### Participant recruitment and sampling

Inclusion criteria for participants were that they were healthcare professionals working in specialist palliative care, had a minimum of 1 year of experience working in specialist palliative care, and had experience of engaging with patients with advanced cancer in specialist palliative care and their caregivers in the decision-making process for patient care. We sought to capture variation in participants’ experiences, and so we did not limit recruitment to specific specialist palliative care sites. We compiled a detailed project flyer (including eligibility criteria and contact details for the research team), which was circulated by a national clinical program for palliative care and by palliative care and hospice support organizations in Ireland. The project flyer was also posted by the research team on social media platforms LinkedIn (an online platform that connects professionals) and X (formerly Twitter). Healthcare professionals who were interested in participating contacted the research team.

The majority of people who contacted the research team upon hearing about the study (70%) participated in the study. Of the people who contacted the research team and who did not participate, three of them consented to participate, but their availability did not align with the scheduling of focus groups despite multiple attempts to organize a time to facilitate their participation. Three others did not reply to the research team after their initial expression of interest and the provision of a participant information leaflet to them. The final two people who expressed interest in the study and who did not participate did not meet the inclusion criteria for the study. Sampling was primarily purposive (Hennink et al. 2020) because we intentionally sought participants based on characteristics and experiences as detailed in the eligibility criteria, to yield information for the topic at hand. Sampling procedures also comprised snowball sampling, whereby a minority of the sample (*n* = 3) were recruited by a participant of the study. Participants who were snowball sampled met the inclusion criteria. A detailed participant information leaflet was provided to each person who contacted the research team and who was eligible to participate. Each person who participated in the study gave informed consent to participate by signing a consent form before participating in the study. Recruitment was carried out by the first author (JH) between February 2025 and May 2025.

### Description of participants

The sample comprised 19 participants (17 women and 2 men) from different professions (nursing, medicine, occupational therapy, physiotherapy, speech and language therapy, medical social work, and nutrition and dietetics). Participants’ time working in specialist palliative care ranged between 3 years and 22 years, and the average time of participants working in specialist palliative care was 10 years. Most participants had worked in a variety of specialist palliative care settings, including hospital and hospice services. Hospice-based experience included a mix of inpatient hospice, day and outpatient hospice, and community-based hospice care. All participants had reached at least a senior and/or specialist grade in their profession. Participants worked in different regions across Ireland. The majority of participants worked in the Dublin region. Table 1 details the characteristics of the participants in full.

**Table 1.** Summary of participants

Participant	Profession	Profession grade	Years working in SPC	Current work setting	Experience in SPC
P1	Nursing	Clinical nurse specialist	11 years	Hospice	Inpatient hospice and community-based hospice care
P2	Nursing	Clinical nurse specialist	6 years	Hospice	Hospital and hospice (including community-based hospice care)
P3	Nursing	Clinical nurse specialist	9 years	Hospital	Hospital-based care
P4	Nursing	Clinical nurse specialist	22 years	Hospital	Hospital-based care
P5	Nursing	Clinical nurse specialist	19 years	Hospice	Hospital and community-based hospice care
P6	Nursing	Clinical nurse specialist	17 years	Hospice	Outpatient and inpatient hospice
P7	Medical Social Work	Senior	4 years	Hospice	Inpatient hospice and community-based hospice care
P8	Occupational Therapy	Senior	6 years	Hospice	Inpatient hospice, outpatient hospice, and community-based hospice care
P9	Speech and Language Therapy	Clinical specialist	10 years	Hospital	Hospital-based care
P10	Nursing	Clinical nurse specialist	18 years	Community	Hospital-based care, inpatient hospice, and community palliative care
P11	Physiotherapy	Senior	4 years	Hospice	Hospice inpatient and daycare
P12	Medicine	Specialist registrar	3 years	Hospice	Hospital and hospice (including inpatient, outpatient, and community-based hospice care)
P13	Medicine	Specialist registrar	4 years	Hospice	Hospital and hospice (including inpatient hospice and community-based hospice care)
P14	Medicine	Specialist registrar	4 years	Hospital	Hospital and hospice (including inpatient, outpatient, and community-based hospice care)
P15	Medicine	Specialist registrar	4 years	Hospital	Hospital and hospice (including inpatient, outpatient, and community-based hospice care)
P16	Occupational Therapy	Senior	20 years	Hospice	Hospice inpatient, hospice day unit, and community-based hospice care
P17	Nutrition and Dietetics	Manager	5 years	Hospice	Inpatient hospice
P18	Medical Social Work	Senior	16 years	Hospice	Inpatient hospice and community-based hospice care
P19	Occupational Therapy	Senior	5 years	Hospice	Hospice inpatient, hospice day unit, and community-based hospice care

SPC = specialist palliative care. Gender: women *n* = 17, men *n*  = 2.

Regions (Ireland): Northeast (*n* = 1), Midlands (*n* = 1), West (*n* = 1), Northwest (*n* = 1), Midwest (*n* = 2), Dublin North (*n* = 7), Dublin South (*n* = 2), Dublin South and surrounding counties (*n* = 3), Dublin West (*n* = 1).

### Data collection

Data collection consisted of 5 focus groups, and each participant participated in one focus group. We chose focus groups as a method for data collection because focus groups allowed participants not only to communicate their viewpoint, but also to understand and clarify their experiences in relation to one another (Tritter and Landstad 2020). A focus group schedule containing open-ended questions was developed by the research team. Questions were formulated to meet the aims of the study. The questions were directly informed by a systematic review on concordance and discordance in decision-making between patients and caregivers in palliative care led by the last author (Mulcahy Symmons et al. 2023), our prior work on healthcare professionals’ perceived role in specialist palliative care in terms of supporting patients and caregivers in end-of-life care decision-making (Featherstone et al. 2025), relational frameworks proposed for end-of-life care (Borgstrom 2015; Gomez-Virseda et al. 2019), and by a systematic review conducted on shared decision-making in cancer palliative care (Rabben et al. 2024). The schedule was shared with participants prior to their focus group. Table 2 details the questions in the focus group schedule.

**Table 2.** Focus group schedule

Q.1
a) From your experience in the field, what kind of decisions about treatment and care are the most challenging for patients with advanced cancer? And why? What kind of decisions are the least challenging for patients with advanced cancer? And why?
b) Family caregivers are commonly involved in discussions about the patient’s treatment and care. What do you feel is most (and least) challenging for family caregivers (of people with advanced cancer) when involved in these discussions? And why?
Q.2
In palliative care, patients with advanced cancer and their family caregivers sometimes differ in their preferences for patient treatment and care. Do you feel that this is or can be problematic for the patient and their family caregiver? If so, why? If not, why not?
Q.3
What are/might be key challenges for you in your day-to-day practice when patients (with advanced cancer) make choices about their treatment and care that do not align with their family caregiver’s wishes? And why?
Q.4
What best helps the patient (with advanced cancer) and their family caregiver manage any disagreement they may have between them about treatment and care for the patient?
Before a decision(s) is made about treatment and care.
After a decision(s) is made about treatment and care.
Q.5
What knowledge, skills, and resources do you rely on to help the patient (with advanced cancer) and family caregiver resolve their disagreements or at least accommodate each other’s preferences?
Q.6
Are there skills and resources that you still need to develop and/or obtain to help the patient (with advanced cancer) and family caregiver resolve their disagreements or at least accommodate each other’s preferences? If so, what might these be?
Q.7
What do you think is most effective to help the patient and family caregiver manage conflict or disagreement in the advanced stages of the patient’s illness? And why?
Q.8
Patients in palliative care often make decisions about their treatment and care with their family caregiver. Do you feel that this has any impact on the patient’s autonomy to make choices about their care? If so, in what way? If you feel it does not have an impact, can you explain why?
Q.9
From an ethics point of view, what do you think is important to consider when decision-making is shared between the patient and their family caregiver?
Q.10
Is there anything else that you would like to say before the focus group comes to a close?

The focus groups were organized by the first author (JH), and each focus group was conducted by the last author (GF) as moderator. The first author compiled field notes during and after each focus group to help contextualize the data for analysis. All focus groups took place online using the video conferencing platform Zoom. Online focus groups were chosen given the different geographical settings of participants and to make it more feasible to schedule a focus group at a time that was suitable for all participants of a focus group (Shelton and Jones 2022). Member checking of the data was done by the last author via confirming participants’ perspectives and seeking clarifications from them as appropriate during their focus group. All focus groups were recorded via Zoom and then transcribed by the first author. The focus groups were conducted between March 2025 and May 2025. The duration of focus groups ranged between 53 minutes and 71 minutes, and the average duration of the focus groups was 64 minutes.

### Data analysis

The data were analyzed using thematic analysis (Braun and Clarke 2021). In the initial stage of the analysis, the focus group transcripts were read multiple times to ensure familiarization with the data. This was followed by the generation of initial codes, which entailed labelling salient data with a name that best reflected the data being coded. As coding proceeded, similar codes were aggregated to form tentative themes that explained participants’ perspectives on discordance between patients with advanced cancer and caregivers in the decision-making process, including barriers and facilitators for patients, caregivers, and participants themselves in terms of managing patient and caregiver discordance. We then refocused the analysis at the theme level by closely reviewing the themes to ensure that all data relevant to them were accounted for. The final stage of analysis comprised a full delineation and naming of the themes. The first author (JH) led the analysis and was supported by the last author (GF) at all stages of the analysis. Coding was cross-checked between the first and last authors to promote trustworthiness (i.e., rigor) in the analysis. The software program NVivo 14 (Lumivero 2023) was used as a tool to code the data.

## Findings

Here, we report key themes that depict participants’ perspectives. The first section of the results details what participants felt aided or challenged patients with advanced cancer and their caregivers to manage their discordance in decision-making. The second section details what participants perceived as helpful or challenging for themselves to manage patient and caregiver discordance in decision-making. All participant quotations below have been pseudonymized and are tagged with a participant ID code (i.e., P1, P2) as shown in Table 1.

## Section 1

### Trust between patient, caregiver, and healthcare professional

Participants reported that trust between the patient, caregiver, and healthcare professional was key to patients’ and caregivers’ own ability to manage their discordance in decision-making:

*I think it makes it better in the long run if people feel that they can trust each other and that we all can have those conversations … so that you can get to a point where they can agree* (P6, focus group 2).

Central to trust between the patient, caregiver, and healthcare professional was a shared openness between the patient, caregiver, and healthcare professional. For example, in relation to making decisions about cancer treatments, P8 (focus group 3) commented:

*The key point is that it [decision] is informed … that everyone is hearing that information, and everyone is hearing about the side effects, and everyone is hearing the benefits as well, and that it is an informed decision by all*.

### Consistent communication between patient, caregiver, and healthcare professional

Participants expressed that consistent communication between the patient, caregiver, and healthcare professional was also necessary for patients and caregivers to manage their discordance in decision-making:

*A lot of decisions can be made on the back of information that probably hasn’t been dissected enough by the two individuals [patient and caregiver] … [but] I think to decipher the actual information to the two* *people and bring them together is needed. It is not just the patient that we are interacting with. It is all the family, and we are trying to manage expectations and differences* (P2, focus group 1).

From the perspective of participants, key to dependable communication between the patient and caregiver, and healthcare professionals, was a genuine attempt to be respectful of each other’s wishes for care. For example, when explaining how patients and caregivers can best navigate discordance about patient place of death, P6 (focus group 2) shared:

*Sometimes people [patients] will very strongly say at the start, I want to die at home. But the families are unsure that they can keep the person at home … You can’t whitewash things or say ‘that’s absolutely what we can do that for you’ because you might not be able to do anything of the sort. And it might not be the case that they can die at home because they have symptom issues that can’t be managed within the home, or the caregiver can’t manage the care … So these conversations need to be smart and respectful between all from the start.*

### Emotional and cognitive burden for both patient and caregiver

Participants perceived that emotional and psychological burden for both the patient and caregiver were significant barriers for patients and caregivers to accommodate each other’s preferences. For example, participants felt that patients’ decisions to opt against treatments that might otherwise prolong the psychological burden of living with metastatic cancer could inadvertently result in psychological distress for their caregivers and make it difficult for patients and caregivers to resolve their differences:

*I think there is the potential for differences that cannot be resolved … When somebody [patient] has persistently declined treatment, but the family really feel that that’s something they should have done. That has the potential to create long-standing burden for the family … maybe a guilt of why they didn’t encourage them to have more treatment … I think sometimes their differences remain unresolved* (P11, focus group 3).

### Preexisting conflict between patient and caregiver

Preexisting conflict between the patient and caregiver was also perceived by participants to be a key barrier for patients and caregivers in terms of navigating and/or resolving their differences in decision-making:

*I think that it is a most stressful time in their lives, all with different opinions, ways of coping … [Yet] sometimes they have grown apart … had disagreements … [and] I think the minuity* *of any disagreement is then sometimes magnified* (P18, focus group 5).

In cases of preexisting conflict, participants indicated that the forced transition for a significant other into a caregiver role could make it more difficult for both the patient and caregiver to accommodate each other’s differences. P3 (focus group 1) explained:

*I have been in several situations where the patient, for example, wanted to stop everything, stop all treatment but the family caregiver did not agree. Situations where the patient was like ‘Oh, can you stop my husband coming in today, I don’t have the energy for us, I don’t have the energy to fight him as well.’ A case I am recalling specifically is a husband who managed her [patient’s] cancer, her treatment and any trials. But to actually have to be her caregiver, was very, very challenging for him.*

## Section 2

### Knowledge and professional experience

All participants agreed that the knowledge and experience gained from engaging directly with patients and caregivers in the decision-making process were instrumental when seeking to help the patient and caregiver manage their discordance in decision-making. Indeed, many participants indicated that experiential learning was equally or in some cases more useful to them than formal training or continued professional development they might otherwise undertake. For example, P1 and P14 disclosed:

*I think that education is obviously important for half of it, but I think if you don’t have the foundation and the experience to build on, I don’t think the education on its own is sufficient* (P1, focus group 1).

*I definitely find the more that I do this, the more comfortable that I feel doing it … Every situation is so nuanced. I don’t think there is any kind of formal communication training that would be better than experience* (P14, focus group 4).

Nonetheless, participants still recognized the value of research and evidence to manage patient and caregiver discordance. As P10 (focus group 3) commented:

*I find that if there is maybe indecision or conflict … if I can bring them back to the rationale for my recommendation, that I can back it up with evidence, I feel people will respond to research and to what other outcomes have been. I don’t think I can ever just give a statement or an opinion without a backup*.

### Resilience and peer support

Having resilience to help the patient and caregiver navigate their differences in decision-making was important for participants. Professional resilience was centered on their ability to accept the potential limitations in their role:

*Compared to other settings [services], you can’t just fix a piece of equipment. In palliative care, there isn’t that fixing so much. It’s helping people on their journey … which I think is a massive thing in palliative care* (P16, focus group 5).

Participants frequently stated the importance of receiving support from colleagues in a multidisciplinary care setting and they strongly agreed that such peer support was essential to decipher how best to address patient and caregiver discordance in decision-making. P7 (focus group 2) explained:

*I think good MDT collaboration is really key. If we are supporting each other and working together, and sharing the same message with people, just even having the support to debrief around things. I think that all really helps.*

### Ethical and legal uncertainties

Participants disclosed that they were sometimes conflicted about the ethical connotations of assisting patients and caregivers resolve their differences concerning patient treatment and care. For example, some participants felt that helping the patient and caregiver manage discordance could potentially undermine patient autonomy in decision-making. For example, P13 (focus group 4) voiced:

*I think at the end of the day we are very much focused on patient autonomy and what is the most important thing to the patient. If they have been aligned with their caregiver all the way along and their caregiver has been their person who has been representing their wishes, that is fine. But I think that’s a different scenario where from the very get go, there is discordance, then that poses ethical issues.*

Uncertainty with respect to decision-making legislation was also perceived by participants as a barrier to helping patients and caregivers manage discordance. Participants reported, for the most part, a lack of familiarity with assisted decision-making legislation in Ireland (i.e., Assisted Decision-Making [Capacity] Act) and an insufficient understanding of its impact or potential impact on decision-making in palliative care:

*Obviously, I have done reading around the Act but I still don’t know if I feel comfortable applying it in a clinical scenario … I think if I had to see a consult this morning where that was involved, I would be worried that I wasn’t doing it properly … I am still not comfortable with the Act* (P14, focus group 4).

### Conflict in relations between patient and caregiver

Overall, participants felt that conflict in relations between the patient and caregiver hindered their own ability to help the patient and caregiver manage their discordance in decision-making. Assisting the patient and caregiver to manage any disagreement required careful deliberation on the part of participants. However, relational conflict between the patient and their caregivers could, in some cases, nullify their efforts to deliberate. For example, P17 and P14 disclosed:

*[With conflict] I am unsure at what point do we ask people “How do you want to make decisions? Who would you like to be involved? … I am not sure* (P17, focus group 5).

I think as time goes on and I have more experience, I see sometimes there’s maybe about 5% of the really complex cases where you just will not get the patient and the family caregiver on the same page (P14, focus group 4).

## Discussion

To our knowledge, this study is the first to investigate specialist palliative care healthcare professionals’ perspectives on what facilitates or hinders patients’ (with advanced cancer) and caregivers’ ability to deal with their differences concerning patient treatment and care. In our study, from the perspective of healthcare professionals, open and trusting relationships between all (i.e., patient, caregiver, and healthcare professional) helped the patient and caregiver manage their discordance. Our findings pertaining to the importance of trust and of open and consistent communication between patients, caregivers, and healthcare professionals in the decision-making process for patients and caregivers resonate with other palliative care studies (McCauley et al. 2023; Fagan et al. 2024; Featherstone et al. 2025), including cancer-specific studies in palliative care (Ribi et al. 2022; Rosa et al. 2023).

Emotional and psychological burden for patients and caregivers in palliative care is well documented (Diaz-Frutos et al. 2016; Oechsle et al. 2019). Our findings pertaining to emotional burden are novel in the context that discordance in decision-making between patients and caregivers can (from the perspective of healthcare professionals) exacerbate emotional distress for both patients and caregivers and make it more difficult for them to resolve their differences. Moreover, we identified that relational conflict (including preexisting conflict) and associated distress between patients and caregivers can make it challenging not only for patients and caregivers but also for healthcare professionals to manage patient and caregiver discordance in decision-making.

We found that healthcare professionals in specialist palliative care felt it necessary to be resilient when helping patients with advanced cancer and their caregivers manage their differences in the decision-making process for patient treatment and care. A scoping review on resilience building in palliative care professionals (Yongpraderm et al. 2025) identified that peer and organizational support were found to be supporting elements of resilience-building programs. We identified that, in addition to support from others, healthcare professionals’ resilience centered primarily on their ability to accept the potential limitations of their role in patient and caregiver decision-making.

As reported, participants placed significant weight on experiential learning when assisting patients and caregivers in managing their discordance in decision-making. These findings are consistent with other studies in palliative care, which have focused on the role of healthcare professionals in the decision-making process for patients with advanced cancer. Luna-Meza et al. (2021) identified that healthcare professionals relied primarily on their professional experience when they approached end-of-life decision-making with patients who had advanced cancer. Rabben et al. (2025) found that healthcare professionals needed to connect their formal education with experiential knowledge when guiding patients and caregivers in decision-making for advanced cancer treatments and palliative care. Our findings support the significance of experiential knowledge for healthcare professionals tasked with managing discordance in decision-making between patients with advanced cancer in palliative care and their caregivers. End-of-life communication interventions in advanced cancer that fully incorporate healthcare professionals’ experiential learning could serve to be effective for managing patient and caregiver discordance in decision-making.

Participants were conflicted by the possibility that helping the patient with advanced cancer and the caregiver negotiate or resolve their differences in the decision-making process could potentially constrain patient autonomy. There is already much debate as to what constitutes patient autonomy in palliative care (Wilson et al. 2014; Houska and Loučka 2019). Einstein et al. (2015) reported that healthcare professionals’ conflicting feelings about autonomy for patients with advanced cancer impeded their ability to make recommendations about patient do-not-resuscitate status. The findings of our study point to the importance of deciphering in more depth how healthcare professionals in specialist palliative care construct patient autonomy. It is possible that formal training for healthcare professionals in specialist palliative care on legislation concerning patient decision-making could alleviate healthcare professionals’ concerns about patient autonomy when assisting patients and caregivers resolve differences in their preferences for and/or decision-making about patient treatment and care.

### Strengths and limitations

We have identified from the perspective of healthcare professionals in specialist palliative care, key factors that aid or challenge patients with advanced cancer, caregivers, and healthcare professionals themselves, in managing patient and caregiver discordance in decision-making for patient treatment and care. The sample size was not large enough to ensure robust representation across each profession, and we did not manage to capture some other key professions in palliative care (e.g., psychology and pharmacy). A larger sample size to allow for robust representation across each profession, combined with recruitment of participants from professions that we did not capture, would have served to expand and further interrogate our findings. However, we managed to recruit healthcare professionals with sufficient variation to enable transferability of our findings to both similar and different contexts in specialist palliative care.

## Conclusions

Our study provides valuable insights into healthcare professionals’ perspectives on discordance between patients with advanced cancer in receipt of specialist palliative care and their caregivers. Participants valued patient autonomy, but also the need to engage fully with caregivers when present in the decision-making process. Different perspectives exist in palliative care with respect to the degree to which caregivers should be involved in the decision-making process for patient treatment and care (Payne and Grande 2013; Blackler 2016). Our findings suggest that healthcare professionals who specialize in the delivery of palliative care to patients with advanced cancer need to communicate as effectively with caregivers as they do with patients when helping the patient and caregiver resolve their differences pertaining to patient treatment and care. Healthcare professionals’ preference to consult with caregivers can run contrary to patients’ preference not to involve caregivers directly in decision-making about the patient’s treatments and supportive care. Further exploratory research is needed to decipher how healthcare professionals who provide specialist palliative care to patients with advanced cancer can effectively acknowledge and support the caregiver role and still deliver care that is respectful of the patient’s preferences.

The findings point to the importance of healthcare professionals being cognizant of the emotional burden shared between patients with advanced cancer in palliative care and their caregivers. We know that deterioration in the emotional health of people with cancer and who have palliative care needs can exacerbate emotional burden for their caregiver (Krug et al. 2016). In our study, participants felt that discordance between the patient and caregiver in decision-making exacerbated distress for both the patient and caregiver. It is possible that interventions focused on assisting patients with advanced cancer and their caregivers in managing their differences in decision-making could serve to alleviate emotional burden for both the patient and caregiver.
